# Analysis of patient-specific stimulation with segmented leads in the subthalamic nucleus

**DOI:** 10.1371/journal.pone.0217985

**Published:** 2019-06-19

**Authors:** T. A. Khoa Nguyen, Milan Djilas, Andreas Nowacki, André Mercanzini, Michael Schüpbach, Philipp Renaud, Claudio Pollo

**Affiliations:** 1 Department of Neurosurgery, Bern University Hospital, Bern, Switzerland; 2 Aleva Neurotherapeutics, Lausanne, Switzerland; 3 Microsystems Laboratory, School of Engineering, EPF Lausanne, Lausanne, Switzerland; 4 Department of Neurology, Bern University Hospital, Bern, Switzerland; Oslo Universitetssykehus, NORWAY

## Abstract

**Objective:**

Segmented deep brain stimulation leads in the subthalamic nucleus have shown to increase therapeutic window using directional stimulation. However, it is not fully understood how these segmented leads with reduced electrode size modify the volume of tissue activated (VTA) and how this in turn relates with clinically observed therapeutic and side effect currents. Here, we investigated the differences between directional and omnidirectional stimulation and associated VTAs with patient-specific therapeutic and side effect currents for the two stimulation modes.

**Approach:**

Nine patients with Parkinson’s disease underwent DBS implantation in the subthalamic nucleus. Therapeutic and side effect currents were identified intraoperatively with a segmented lead using directional and omnidirectional stimulation (these current thresholds were assessed in a blinded fashion). The electric field around the lead was simulated with a finite-element model for a range of stimulation currents for both stimulation modes. VTAs were estimated from the electric field by numerical differentiation and thresholding. Then for each patient, the VTAs for given therapeutic and side effect currents were projected onto the patient-specific subthalamic nucleus and lead position.

**Results:**

Stimulation with segmented leads with reduced electrode size was associated with a significant reduction of VTA and a significant increase of radial distance in the best direction of stimulation. While beneficial effects were associated with activation volumes confined within the anatomical boundaries of the subthalamic nucleus at therapeutic currents, side effects were associated with activation volumes spreading beyond the nucleus’ boundaries.

**Significance:**

The clinical benefits of segmented leads are likely to be obtained by a VTA confined within the subthalamic nucleus and a larger radial distance in the best stimulation direction, while steering the VTA away from unwanted fiber tracts outside the nucleus. Applying the same concepts at a larger scale and in chronically implanted patients may help to predict the best stimulation area.

## Introduction

Deep brain stimulation (DBS) of the subthalamic nucleus (STN) has demonstrated efficacy in treating motor symptoms of Parkinson's disease [1,2]. It delivers electrical pulses at high stimulation frequencies to pathogenic brain areas using leads with four (Medtronic Activa, Abbott St Jude Libra) to eight (Boston Scientific Vercise) ring electrodes. Most implanted DBS systems provide current delivery in all directions around the electrode. This omnidirectional mode has shown to significantly improve motor symptoms, but unintended stimulation of surrounding anatomical structures can induce disabling side effects such as tonic muscular contraction, dysarthria, conjugate eye deviation, paresthesia, or gait imbalance [3–5]. More specifically, unintended stimulation of the non-motor parts of the STN may cause behavioral impairments and limbic side effects such as depression and impulsivity [6–8]. To reduce or avoid these side effects, one can decrease the stimulation amplitude. However, this typically reduces treatment efficacy as less stimulation is directed towards the target structure, which is the dorsolateral, or motor part, of the STN [2].

To optimize DBS therapy, millimeter to sub-millimeter targeting accuracy in the motor part of the STN may be needed [9–11]. Despite careful stereotactic targeting, targeting errors may occur due to imaging inaccuracy, imperfect visualization of target structures, mechanical stereotactic errors, intraoperative brain shift due to leakage of cerebrospinal fluid [12–14] and patient-specific anatomy placement errors. These errors amount to about 1–2 mm with current stereotactic procedures [15,16], and therefore may produce current spread into adjacent structures and the appearance of side effects [2].

Segmented leads with reduced electrode size are a promising option to improve DBS therapy. Typical ring electrodes are 1.3 mm in diameter and 1.5 mm in height with a surface area of about 6 mm^2^. Microfabrication techniques allow for thin-film based segmented DBS leads providing electrodes of reduced size and surface areas of about 1 mm^2^ [17–21]. Such segmented leads allow for *directional* stimulation. They showed to increase therapeutic windows intraoperatively by requiring smaller current amplitudes to obtain therapeutic effects compared with omnidirectional stimulation [22,23]. These results were confirmed in chronically implanted patients in additional centers [24,25].

To better understand the mechanisms of DBS, finite-element models of traditional ring electrodes have greatly contributed to an understanding of the volume of tissue activated [26–29]. These activation volumes and their positions in the STN have been associated with therapeutic and/ or side effects. However, how *segmented* leads with reduced electrode size modify these volumes and how this correlates with the clinical observations of changed therapeutic and side effect thresholds is not fully understood.

In the present study, we investigated the effects of omnidirectional and directional stimulation of the STN through a computational analysis of volume of tissue activated (VTA) and correlated these with patient-specific therapeutic and side effects current thresholds.

## Material and methods

### Segmented DBS lead

**Fig 1:**
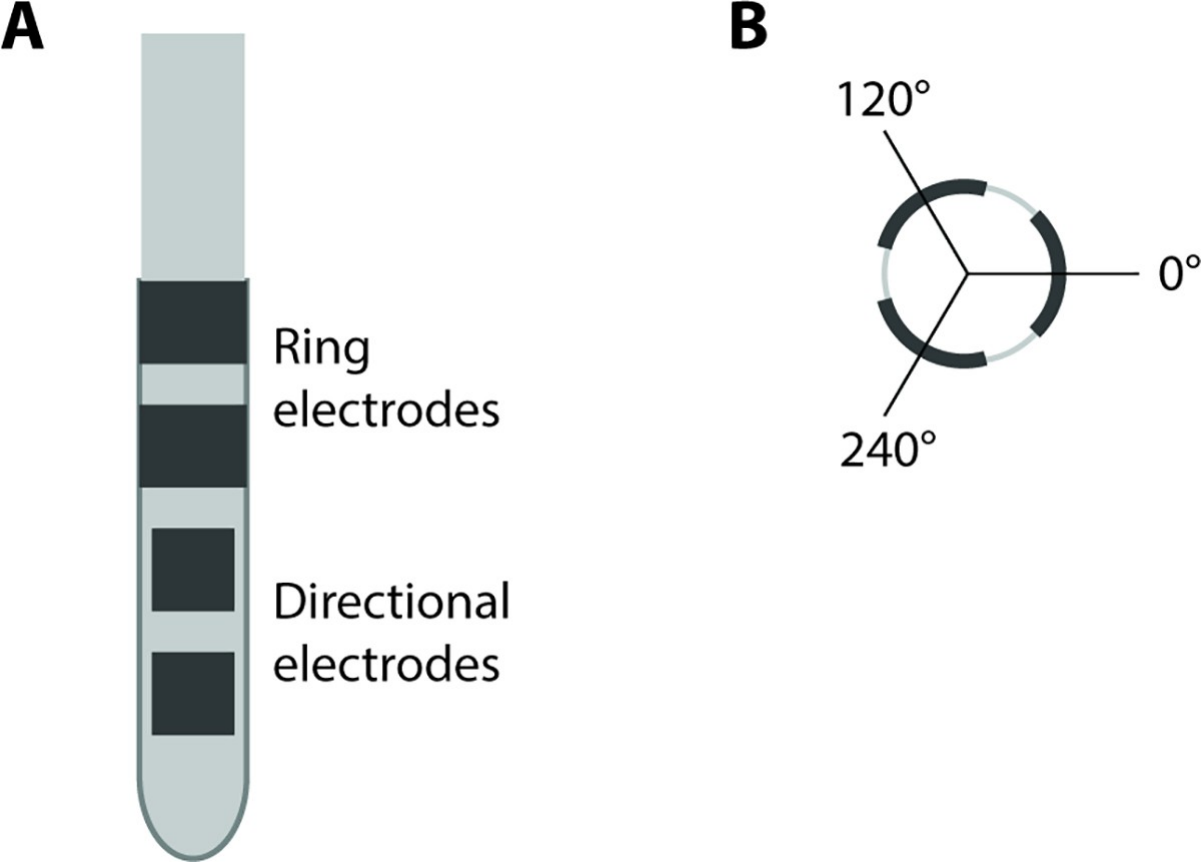
Design of segmented lead. (A) The segmented lead had a diameter of 1.3 mm. It consisted of eight electrodes in total: two levels with three directional electrodes each (size 1 x 1 mm^2^) and two levels of ring electrodes (size 1 x 4.1 mm^2^). The distance between electrode levels was 0.5 mm. (B) The directional electrodes were facing 0, 120 and 240° and 0° was facing medial.

A segmented DBS lead with six directional and two ring electrodes was used in this study (directSTN Acute, Aleva Neurotherapeutics, Lausanne, Switzerland, Fig 1). The lead’s directional electrodes were on the two most distant electrode levels and only the most distant level was used in this study. The two ring electrodes made up the two proximal electrode levels. The directional electrodes were each 1 mm x 1 mm (surface area of 1 mm^2^), and the distance between each level was 0.5 mm. The three directional electrodes were distributed at 0°, 120° and 240°, respectively. The electrode at 0° was marked with an imprinted black line all along the lead to help the surgeon orient it during insertion.

### Patients, stimulation pattern and intraoperative assessments

Nine patients with Parkinson’s disease underwent DBS of the subthalamic nucleus (three female, age 32–74, median 69 years) and were included in this study at the University Hospital of Bern. The study conformed to the Good Clinical Practice guidelines and the International Organization for Standardization 14155 standard. The protocol was approved by the ethics committee of the Canton of Bern and by the Swiss Competent Authority. All patients provided written informed consent. The patients are listed in Table 1 and represented a subset of the cohort of this previous study [23]. The study herein compares omnidirectional and directional VTAs in general. It further focuses on patient-specific stimulation currents and adds VTA projections for each patient.

**Table 1:** Patient information. Patient information with therapeutic and side effect currents. All patients with Parkinson’s disease and rigidity symptoms.

**Patient number/** **hemi-** **sphere**	**Gender**	**Age**	**1**^**st**^ **best direction**				**Omnidirectional**		
			Direction	Therapeutic current (mA)	Side effect current (mA)	Side effect	Therapeutic current (mA)	Side effect current (mA)	Side effect
1 Right	Male	72	PL	0.4	3.3	Dysarthria	0.9	4*	*No side effect
2 Left	Male	52	M	0.9	3.5*	*No side effect	1.2	2.7	Lip contraction
3 Left	Male	74	AL	0.5	3.5*	*No side effect	1.2	3.9	Parasthesia
4 Left	Male	42	AL	1.0	2.7	Dysarthria	1.8	2.5	Dysarthria
5 Right	Female	69	AL	0.5	3.5*	*No side effect	1.1	3.0	Paresthesia
6 Left	Female	70	AL	0.6	2.0	Dysarthria	0.8	1.1	Dysarthria
7 Left	Male	55	AL	0.4	2.5	Dysarthria	1.2	2.5	Dysarthria
8 Left	Female	70	M	1.0	3.3	Dysarthria	1.5	3.3	Dysarthria
9 Right	Male	32	M	0.6	3.3	Lip contraction	0.8	2.7	Lip contraction

Directions: M–medial; AL–antero-lateral; PL–postero-lateral.

An asterisk * denotes cases where no sustained side effect was evoked at the maximum current amplitude listed in the Table.

The surgery was performed as described previously [23]. Briefly, preoperative T1 and T2-weighted magnetic resonance images (3 T, 1 mm x1 mm x 1 mm) were coregistered with stereotactic computer tomography for planning. The patients were implanted under local anesthesia under stereotactic conditions (Leksell frame, Elekta). First, microelectrode recording and macrostimulation (FHC, Bowdoin, ME, USA and Leadpoint, Medtronic, Minneapolis, MN, USA) were performed to localize the STN and to identify the trajectory and depth for permanent implantation.

Second, the segmented lead was inserted with the most distal electrode level at the same depth as intended for the permanent lead (i.e., systematically at 2 mm after electrophysiologically determined STN entry). The depth for the segmented lead was confirmed intraoperatively with fluoroscopy. The segmented lead’s directional electrode at 0° was systematically oriented medially using the marker line on the lead (the surgeon inserted the lead without rotation). The second directional electrode was therefore oriented antero-laterally (120°), and the third directional electrode was oriented postero-laterally (240°).

Third, the segmented lead was used for intraoperative clinical testing. The lead was connected to an external neurostimulator (Osiris Stimulators, Model 504196, Inomed GmbH, Emmendingen, Germany). This neurostimulator had multiple independent current-driven sources and was able to pulse any directional electrode or a combination of directional electrodes through a custom user interface. Concomitant use of the three directional electrodes on the same level was defined as omnidirectional stimulation. Monopolar, cathodic monophasic pulses with a phase width of 90 μs and frequency of 130 Hz were used. A metal plate in the subclavicular area was used as distant current return (similar to the area for the implantable pulse generator). The stimulation configuration (i.e., directional or omnidirectional stimulation, which directional electrode) was programmed randomly by a separate operator. Therefore the patient, the neurosurgeon and the neurologist assessing the clinical effects were blinded to the stimulation configuration.

Clinical effects were assessed intraoperatively through the rigidity of the patient’s hand by the same neurologist (M Schupbach). Before stimulation onset, baseline rigidity was evaluated on a five-point rating scale (0 –no rigidity, 4 –highest rigidity). The stimulation current was increased in 0.2 mA steps and the patient was tested until the current threshold with *full effect* on rigidity was reached (value 0 on the described rating scale). This was defined as therapeutic current. The stimulation current was then further increased until a sustained side effect was observed. This was defined as side effect current. Side effects included sustained paresthesia, dysarthria, or focal muscular contraction. The maximally allowed current amplitude was 3.3 mA per directional electrode to ensure safe stimulation below the charge density limit of 30 μC/cm^2^[30,31].

Finally, the segmented lead was removed and replaced with the permanent lead (Medtronic, Minneapolis, MN, USA, model 3389) using the same guide tube. All clinical assessments performed with the segmented lead were performed only in the first hemisphere operated on. The hemispheres are listed with the patients in Table 1.

### Computation of volume of tissue activated and radial distance

A finite-element model was used to simulate the electrical field and to estimate the volume of tissue activated (VTA). The 3D geometry of the segmented lead was imported from Dassault Systems Solidworks 2016 into COMSOL Multiphysics v4.0. The perielectrode domain surrounding the lead was modeled with a thickness of 0.2 mm. The grey matter beyond was represented by a cylinder with a 10 mm radius to mimic placement in the STN and assumed a homogenous isotropic medium (grey matter). The electrical conductivity of the perielectrode space was set to 0.125 S/m, and to 0.3 S/m for the outer grey matter [11,26]. All boundary conditions for the electrical currents physics module were set to insulation, except for the stimulating electrodes, that were configured as current source terminals, and the outer brain region cylinders, that were configured as electrical ground. The electric field was computed from solving the Laplace equation in COMSOL Multiphysics [11].

The volume of tissue activated was then estimated from the electric field with the method presented by Buhlmann et al. [32], who used a segmented lead for their simulation study with similar characteristics as the lead herein. The potential values around the electrode were arranged into a 3D matrix with a spatial resolution of 0.025 mm and were exported to Matlab 2016b (The MathWorks, Natick, MA, USA). This matrix was then numerically differentiated twice by spatial difference calculation in the x, y, and z directions to define one Hessian matrix for each point in space. To specify the maximal second derivative in any direction, the eigenvectors of each Hessian matrix and the corresponding eigenvalues were calculated. The greatest eigenvalue with the corresponding eigenvector gave the magnitude and the direction of the maximum curvature of the electric field. Activation of neural tissue was considered when the maximal eigenvalue exceeded the activation threshold of 26.66 V/cm^2^ as used in [32]. The use of the Hessian matrix as approximation of the neural activating function was described more comprehensively by [21,33].

**Fig 2:**
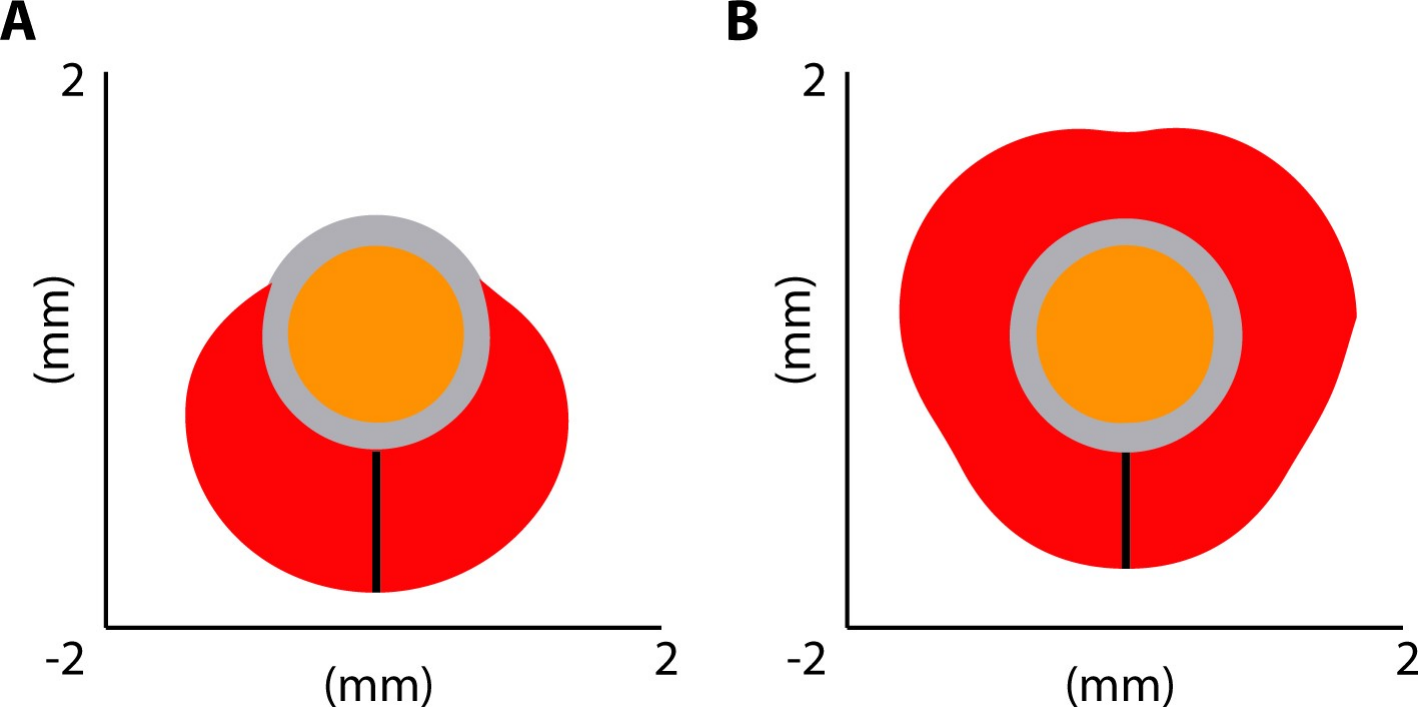
Radial distance illustration. Radial distance shown as black vertical bar in directional (A) and omnidirectional (B) stimulation mode for 1 mA total current. The volume of tissue activated projected onto the axial plane is in red; the lead in orange, and the peri-electrode domain in grey.

We compared VTAs of the segmented lead for two stimulation modes: *omnidirectional* stimulation, in which current was applied to all three directional electrodes on the same level simultaneously, and *directional* stimulation, in which current was applied to one directional electrode only. The VTA was computed for each stimulation mode and for a range of current amplitudes between 0 and 5 mA. To additionally describe the VTA, we calculated the radial distance and defined it as the distance between the surface of the electrode and the limit of the VTA in the axial plane (Fig 2).

### Correlation stimulation current, VTA and observed clinical effects

Several steps were performed to investigate relations between stimulation current, VTA and observed clinical effects. First, the position of the *permanent* lead was determined from postoperative computer tomography images coregistered to preoperative magnetic resonance images (Brainlab iPlan 3.0 Stereotaxy, Brainlab, Munich, Germany). With the stereotactic frame used for implantation, we assume that the tip of the *segmented* lead was at the same depth as the tip of the permanent lead since the same guide tube was used. Second, for each patient the STN was manually segmented in the T2-weighted magnetic resonance images. Then the axial slice at the depth of the directional electrode level was taken. Third, the VTA estimations for therapeutic and side effect currents were projected onto that axial slice to illustrate the outlines of the STN and VTAs.

Finally, the changes in therapeutic current from omnidirectional to best directional stimulation were related to changes in therapeutic window and analyzed for each patient. Similarly, the changes in side effect currents from omnidirectional to best directional stimulation were analyzed.

### Statistical analysis

Therapeutic currents for best directional and omnidirectional stimulation were statistically assessed with a non-parametric paired Wilcoxon signed-rank test, since these current thresholds were not normally distributed (Shapiro-Wilk test). Similarly, the VTAs and radial distances at these therapeutic currents were statistically assessed with a paired Wilcoxon signed-rank test. A p-value of less than 0.05 was considered statistically significant. Side effect currents, the corresponding VTAs and radial distances for the two stimulation modes were also statistically assessed with the same Wilcoxon signed-rank test. Data was analyzed with Matlab 2016b.

## Results

Nine patients were tested intraoperatively with a segmented lead. From the three directional electrodes tested, only the best directional electrode was considered for further analysis to better understand the potential benefits of directional stimulation. This best directional electrode was determined by the largest therapeutic window, i.e., the difference between side effect and therapeutic currents (note that all directional electrodes yielded full effect on rigidity). Therapeutic and side effect currents for directional and omnidirectional stimulation are listed in Table 1.

### Stimulation currents, VTA and radial distance

Therapeutic currents for best directional stimulation were significantly smaller than therapeutic currents for omnidirectional stimulation (median values 0.6 vs 1.2 mA, p = 0.04). In contrast, the side effect currents for both stimulation modes were not significantly different (median values 3.3 vs. 2.7 mA for best directional and omnidirectional stimulation, respectively, p = 0.41).

**Fig 3:**
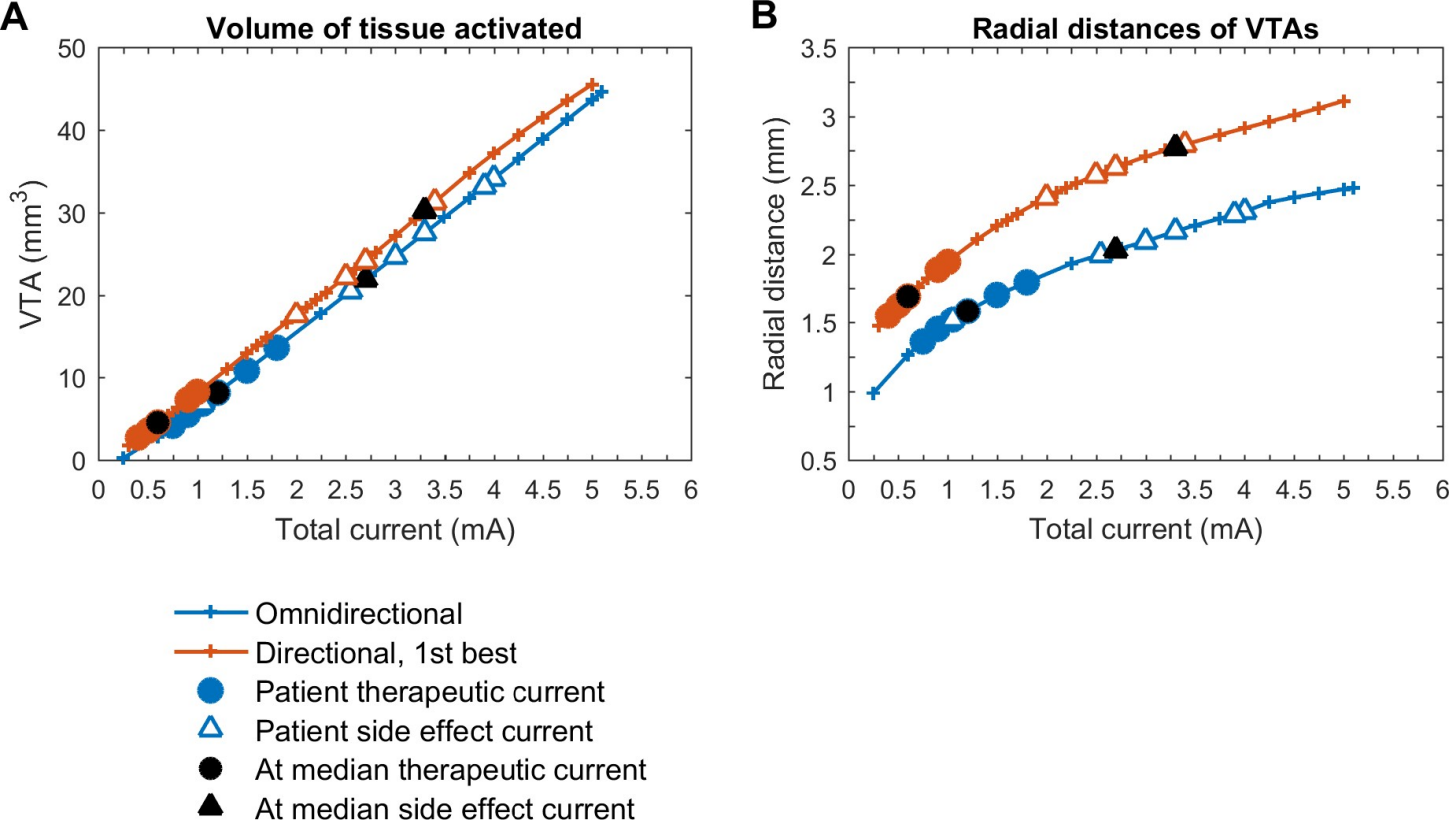
Volume of tissue activated and radial distance. (A) Volume of tissue activated (VTA), (B) radial distance for current amplitudes between 0 and 5 mA.

The volume of VTAs increased linearly with stimulation current for both stimulation modes (Fig 3A). At therapeutic currents, the VTAs with best directional stimulation were about half the VTAs with omnidirectional stimulation (median volumes 4.5 mm^3^ vs. 8.2 mm^3^, p = 0.02). At side effect currents, the VTAs for both stimulation modes were not significantly different, with the VTA in best directional mode slightly larger (median volumes 30.3 mm^3^ vs. 22.0 mm^3^, p = 0.07).

The radial distances of VTAs increased non-linearly with stimulation current for both stimulation modes with larger radial distances for best directional stimulation (Fig 3B). At therapeutic currents, the median radial distance was 1.7 mm for best directional and 1.6 mm for omnidirectional stimulation (p = 0.01). At side effect currents, median radial distances for best directional stimulation were significantly larger than for omnidirectional stimulation (2.8 mm vs. 2.0 mm, p = 0.004).

### Correlation stimulation current, VTA and patient-specific clinical effects

**Fig 4:**
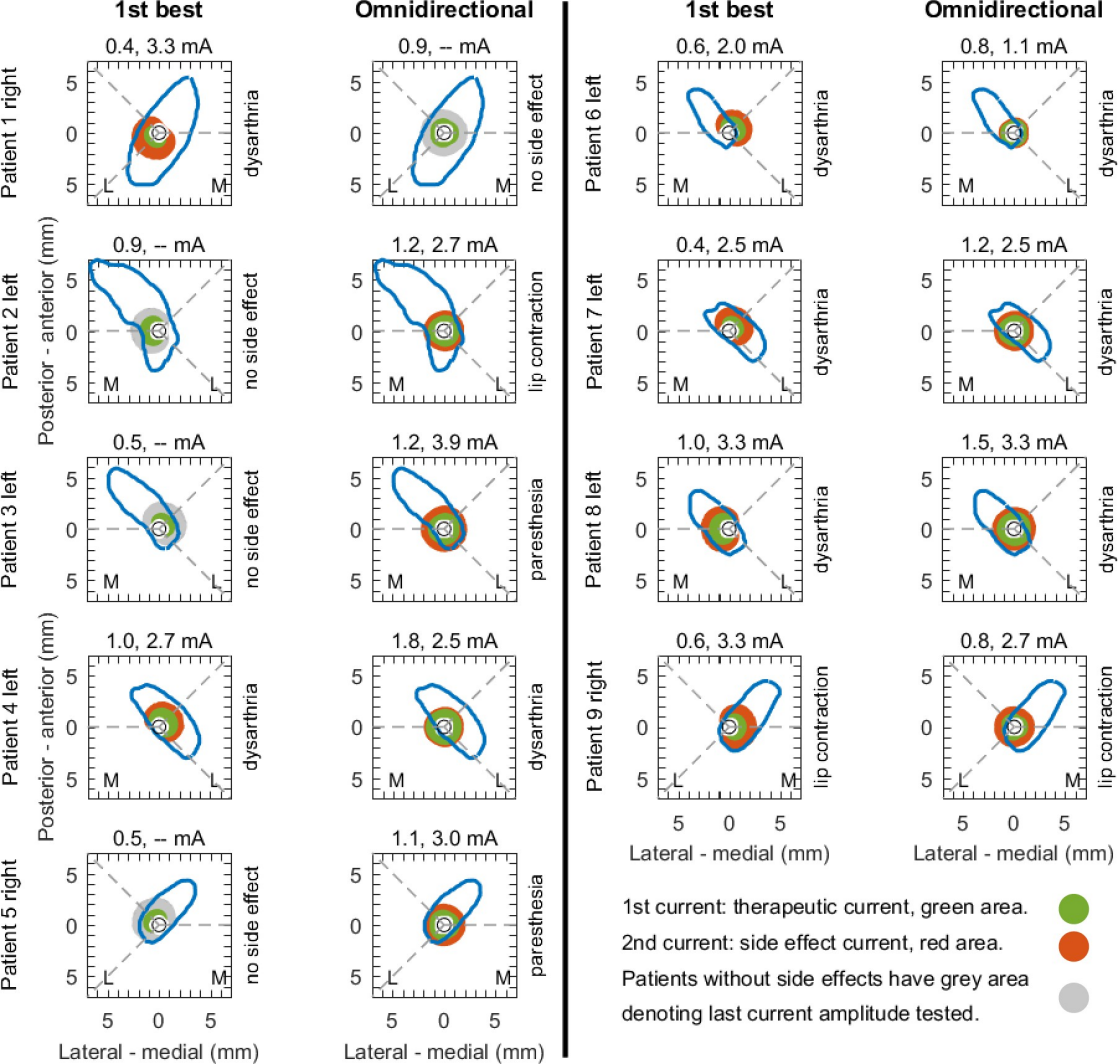
Patient-specific analysis. Projection of the volume of tissue active at the observed therapeutic currents (green) and side effect currents (red) in the nine patients. The volume of tissue activated was projected onto the axially segmented subthalamic nucleus. (The figure is a radiological representation of left and right. The letters ‘M’ and ‘L’ denote medial and lateral for each patient).

At therapeutic currents, VTAs for both stimulation modes were generally confined inside the subthalamic nucleus or maximally 1 mm outside the nucleus (Fig 4). Only patient 4 had the VTA outside the nucleus for omnidirectional stimulation.

At side effect currents, onset and type of side effects were related with VTAs spreading beyond the nucleus' anatomical border (red fields in Fig 4). Capsular side effects such as dysarthria or muscular contraction appeared in several patients and were associated with activation volumes spreading beyond the nucleus into the area of the internal capsule. Interestingly, patients 2, 3 and 5 did not show side effects for best directional stimulation, though the VTAs spread about 1 mm beyond the nucleus’ boundaries (grey fields in Fig 4).

**Fig 5:**
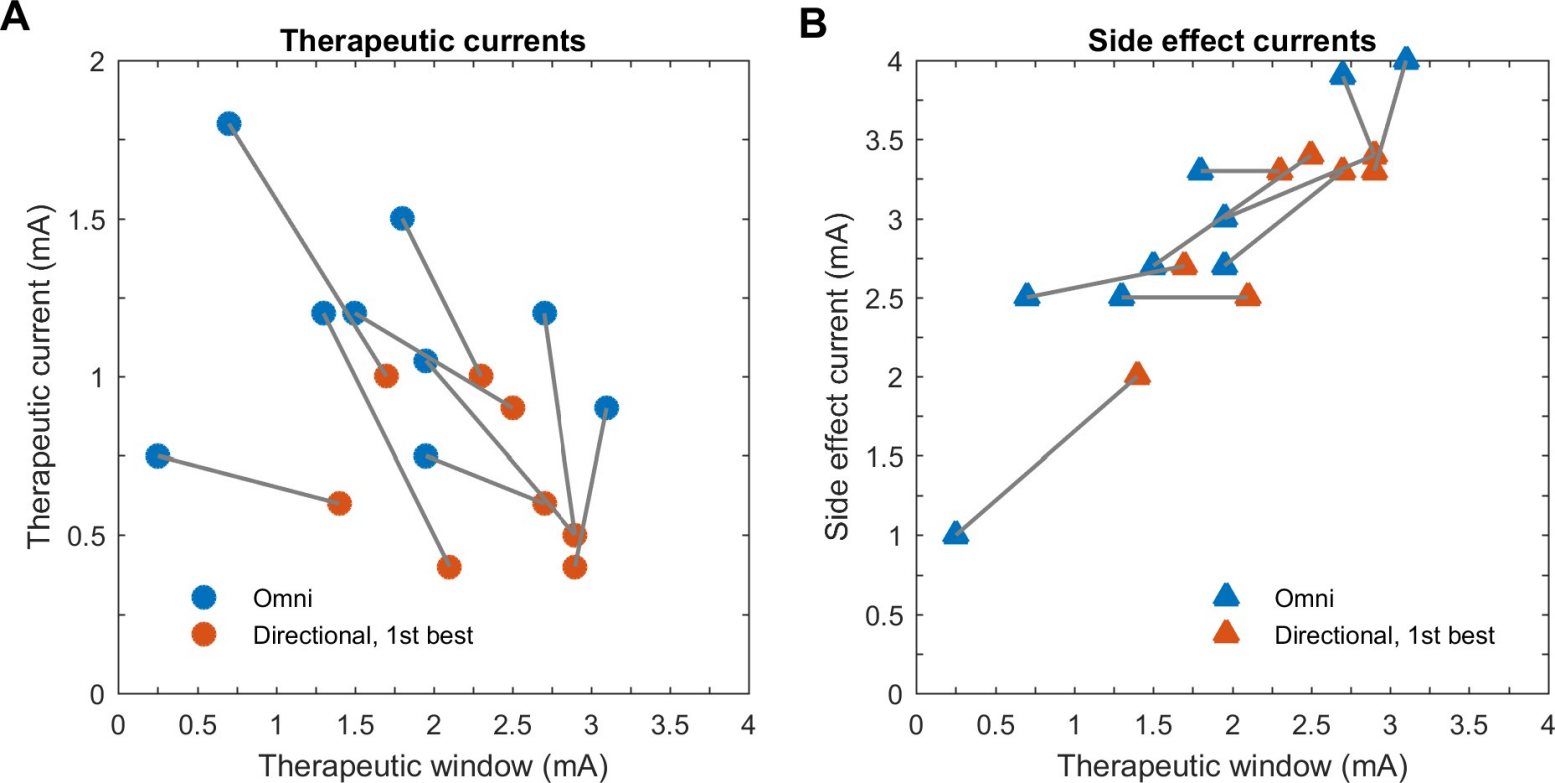
Therapeutic and side effect currents versus therapeutic window. Change of therapeutic (A) and side effect currents (B) over therapeutic window. No correlation analysis was performed given the small patient number.

Therapeutic and side effect currents were different for best directional and omnidirectional stimulation. These changes affected the therapeutic window (Fig 5). We observed that reduced therapeutic currents contributed to an increased therapeutic window in the best directional stimulation in all patients. On the other hand, we observed that only small increases in side effect currents in five patients contributed to the increased therapeutic window (no increase or decrease in four patients). Due to the limited number of patients, we were not able to perform a correlation test.

## Discussion

The present study investigated changes in VTA with *segmented* DBS leads that have smaller sized, but directional electrodes. These changes were related to intraoperative clinical testing in Parkinson’s disease patients that received DBS in the subthalamic nucleus.

At therapeutic currents, our results show that stimulation in the best direction required significantly smaller VTAs than omnidirectional stimulation (three directional electrodes activated simultaneously). Moreover, best directional stimulation generated a significant increase of the radial distance. Taken together, this indicates that best directional stimulation achieved therapeutic effect through deeper tissue penetration in the best direction with smaller VTAs than omnidirectional stimulation.

At side effect currents, VTAs were not significantly different for best directional and omnidirectional stimulation, while radial distance remained significantly different for the two stimulation modes.

### VTA and radial distance

Previous computational DBS studies reported that electrode size may influence the shape and size of the VTA and therefore the occurrence of therapeutic and side effects. These studies also suggested that high precision of lead placement was required to confine VTAs to the target stimulation area [28,29].

Here, we systematically analyzed VTA and stimulation currents for directional and omnidirectional stimulation modes. At therapeutic currents, we obtained a significantly reduced VTA using directional stimulation in the best direction. Reducing the VTA may facilitate targeting areas of the STN that induce therapeutic effects, while avoiding side effects. This hypothesis is sustained by one study that targeted a specific volume to improve motor performance. This volume included the dorsal part of the STN and also white matter dorsal to the nucleus [34]. It was also demonstrated in another study that targeting the dorsal STN can reverse stimulation-induced cognitive impairments in patients [35]. In contrast, stimulation of the ventral STN was associated with postoperative hypomania [7].

We observed an increased radial distance with directional stimulation through the small directional electrode compared to omnidirectional stimulation. A plausible explanation for this observation comes from the finite-element model. The reduction in electrode size with directional electrodes resulted in an increased charge density at the surface of the electrode (charge density is the ratio of injected charge over electrode surface area). Therefore, an increased charge density led to an increased radial distance (tissue penetration) in the best direction of stimulation. For instance, at a total current of 0.5 mA the radial distance for best directional stimulation was 33% larger than for omnidirectional stimulation (1.6 vs. 1.2 mm). At 3 mA total current, the difference in radial distance was 29% (2.7 vs. 2.1 mm). At a clinical level, this phenomenon may correlate with the decreased therapeutic current with directional stimulation as observed in this study.

In another computational study, Wei and Grill suggested that larger electrode size required higher current intensities to obtain the same activation volume [36]. They assumed that reducing electrode size with segmented electrodes would result in better stimulation efficiency. Similarly, Alonso et al. showed in a computational study that electrodes with larger areas had a smaller VTA than electrodes with reduced area [37].

At side effect currents, VTAs for best directional and omnidirectional stimulation were similar. One reason is that median side effect currents were similar for the two stimulation modes (3 vs. 2.7 mA for best directional and omnidirectional stimulation, respectively). Interestingly, the radial distances were different (2.8 mm vs. 2.0 mm), which indicated that best directional stimulation had deeper tissue penetration also at side effect thresholds.

### Correlation stimulation current, VTA and patient-specific clinical effects

We found therapeutic currents to be consistently associated with a VTA confined within the anatomical boundaries of the STN. It seemed that VTAs were partially covering the dorsolateral, motor portion of the STN given that clinical testing was performed at 2 mm after electrophysiologically determined STN entry. This is in agreement with other studies that mapped VTA using omnidirectional stimulation mode with clinical outcomes [34,38]. However, it was not possible to infer from our two-dimensional projections that VTAs were strictly confined to the motor part of the nucleus without spill-over stimulation to non-motor portions.

Another study reported that most patients with the largest improvements in the Unified Parkinsons’s Disease Rating Scale had VTAs *outside* the border of the STN, principally in the Zona Incerta [39]. This apparent discrepancy with our findings might be due to the different metrics and time points of testing. In our study, we exclusively assessed rigidity with an intraoperative rigidity score compared to a postoperative score with the unified rating scale a few months after implantation, which takes other motor symptoms into account. In addition, we used a two-dimensional axial projection compared to the three-dimensional model used by Maks et al. [39].

Our finding that VTAs at side effect current spread beyond the anatomical boundaries of the STN for both stimulation modes seemed plausible and is in agreement with other studies [34,38]. For instance, we observed capsular side effects when VTAs extended into the internal capsule. We assumed that side effects such as muscular contractions or dysarthria were induced by the stimulation of the surrounding cortico-spinal tract (motor dysarthria, muscular contraction), or the dento-rubrothalamic tract (ataxic dysarthria). These tracts are located (antero-)laterally and (postero-)medially to the STN, respectively, when seen on an axial projection.

The enlarged therapeutic windows for best directional stimulation in our study were mostly related with decreased therapeutic currents and to a limited extend with increased side effect currents. In our small cohort, we were not able to find a statistical correlation due to the restricted patient number. Recent chronic studies reported that directional stimulation primarily increased side effect currents [24,25]. On the other hand, DBS of the thalamus for tremor reported decreased therapeutic currents [40]. Considering these different results, there seems so far no clear evidence in STN DBS. Why directional stimulation had smaller therapeutic currents is not fully understood. As discussed above, a deeper penetration of the VTA in the best direction of stimulation (increased VTA radial distance) may be the mechanism underlying this observation. On the other hand increased radial distance may have likewise resulted in lower side effect thresholds for directional stimulation, considering that an increased radial distance in the direction of stimulation may induce side effects at lower currents than omnidirectional stimulation. Here, we believe that avoiding undesirable fiber tracts by steering VTA in a specific direction away from these tracts may have played a more important role.

### Limitations

First, our computational model has several limitations. We used a two-step approach, calculating the electric field with finite-element modeling and then estimating the VTA through numerical differentiation and the Hessian matrix [32]. Since finite-element modeling is computationally expensive, we performed this calculation for one scenario only with the lead placed in grey matter to mimic implantation in the STN. The resulting VTAs at therapeutic and side effect currents were then projected onto the patient-specific lead position inside the STN. These projections were performed in two-dimensional axial slices and assumed the lead to be perpendicular to that slice.

Other approaches for VTA estimation do not require explicit finite-element modeling or a computational axon model. These may be comparable with the method herein [38,41,42]. The accuracy of VTA estimations has been critiqued in detail; therefore more sophisticated three-dimensional models based on field-cable or driving-force pathway activation models should be implemented in future studies[43,44].

Second, this study was performed during the acute phase of lead insertion. The induced microlesional effect and/ or possible surrounding reactive tissue edema may alter the electrical properties of the surrounding tissue. This in turn may change in a more chronic situation, as a progressive increase of the impedance at the electrode-tissue interface has been reported [45]. Therefore, observations made in this acute phase may change over time. Nevertheless, we performed this study using a constant current-based pulse generator, measuring currents delivered to the tissue, therefore independently from impedance. The possible acute modification of the tissue impedance may modify the VTA over time, as the calculations are dependent on tissue conductivities [46]. However, we believe that tissue conductivity properties remained at similar levels during the clinical testing of the segmented lead as this took place after microelectrode recording and macrostimulation to localize the STN (approx. thirty minutes).

Third, determination of current thresholds was solely based on rigidity reduction. This measured only one aspect of PD at a very limited time point and did not take into account other beneficial stimulation effects. Intraoperatively, rigidity is a clinical sign that can be assessed reliably and rapidly. In contrast, bradykinesia can only be assessed over a longer time-frame, while tremor is fluctuating and strongly influenced by emotion and therefore less reliable. Thus assessing rigidity reflects the current clinical standard to assess the most effective clinical contact for long-term stimulation [47].

Fourth, the orientation of the segmented lead was not controlled through imaging intraoperatively. Though we carefully inserted the segmented with its marker line facing medial, various methods exist to determine the orientation of a segmented lead and will be used in future studies [48–50].

Finally, our study was conducted on a limited number of patients. Though we were not able to show statistically significant results regarding the correlation between therapeutic, side effect currents and therapeutic window, we found it useful to show the correlation between the VTA and the anatomical boundaries of the STN in patient-specific situations. Further studies with a high number of chronically implanted patients and more refined computational methods are therefore warranted to confirm these results. This concept could open the door for studies to help predict the best stimulation area using algorithms that scan and evaluate the extended stimulation parameter space for segmented leads [51–53].

## Conclusion

Deep brain stimulation of the STN performed with segmented leads and reduced electrode size resulted in a decreased VTA and increased radial distance at therapeutic currents. Moreover, VTAs were consistently confined inside the anatomical boundaries of the STN at therapeutic currents, whereas they predominantly spread beyond the STN at side effect currents in the direction of undesired fiber tracts. The clinical benefits of directional stimulation are likely to be obtained through a VTA confined within the STN, while steering VTA away from unwanted fiber tracts outside the nucleus. Applying the same concepts at a larger scale and in chronically implanted patients may help to predict the best stimulation area according to specific symptoms using segmented leads.
